# Visual Navigation for Recovering an AUV by Another AUV in Shallow Water

**DOI:** 10.3390/s19081889

**Published:** 2019-04-20

**Authors:** Shuang Liu, Hongli Xu, Yang Lin, Lei Gao

**Affiliations:** 1 State Key Laboratory of Robotics, Shenyang Institute of Automation, Chinese Academy of Sciences, Shenyang 110016, China; xhl@sia.cn (H.X.); liny@sia.cn (Y.L.); gaolei1@sia.cn (L.G.); 2 Institutes for Robotics and Intelligent Manufacturing, Chinese Academy of Sciences, Shenyang 110016, China; 3 University of Chinese Academy of Sciences, Beijing 100049, China

**Keywords:** autonomous underwater vehicle, recovery, detection, pose estimation, visual navigation

## Abstract

Autonomous underwater vehicles (AUVs) play very important roles in underwater missions. However, the reliability of the automated recovery of AUVs has still not been well addressed. We propose a vision-based framework for automatically recovering an AUV by another AUV in shallow water. The proposed framework contains a detection phase for the robust detection of underwater landmarks mounted on the docking station in shallow water and a pose-estimation phase for estimating the pose between AUVs and underwater landmarks. We propose a Laplacian-of-Gaussian-based coarse-to-fine blockwise (LCB) method for the detection of underwater landmarks to overcome ambient light and nonuniform spreading, which are the two main problems in shallow water. We propose a novel method for pose estimation in practical cases where landmarks are broken or covered by biofouling. In the experiments, we show that our proposed LCB method outperforms the state-of-the-art method in terms of remote landmark detection. We then combine our proposed vision-based framework with acoustic sensors in field experiments to demonstrate its effectiveness in the automated recovery of AUVs.

## 1. Introduction

Autonomous underwater vehicles (AUVs) are of great interest to oceanographers and navies for marine scientific research and reconnaissance due to their wide scope of activity and high efficiency. However, long-duration missions struggle with the limited on-board energy and storage capability of AUVs. The autonomous recovery of AUVs is highly desirable for recharging, data transfer, and relaunch. Various recovery systems have been proposed by previous works, and they usually contain a docking station. Docking stations can either be stationary [1,2,3,4], such as landed on the seafloor, or mobile [5,6,7,8,9,10], such as carried by Unmanned Surface Vehicles (USVs) [5] and submarines [7,8,9]. Stationary docking stations are able to perform recharging and data transfer for AUVs, but in fixed locations. Mobile docking stations are more flexible. They can not only recharge and transfer data almost anywhere, but also take AUVs back to home harbors. All recovery systems aim at the precise physical contact between docking stations and AUVs. Precise contact can either be passive or active. Passive contact is to capture AUVs by using custom mechanisms. An AUV was captured by a robotic manipulator when it is in the docking envelope of submarines in [9]. Active contact means that AUVs actively connect to docking stations with the help of custom mechanisms. A common kind of active contact is that AUVs catch a cable [5,8] or pole [11,12,13] with an incorporated custom latch or hook. This form allows AUVs to approach the cable or pole in any direction, but requires additional space to install the mechanism on the AUVs, which limits the wide application to a range of AUVs. The other kind of active contact is using funnel-like docking stations [1,3,14,15,16]. Funnel-like docking stations allow for trajectory errors during docking without much refitting on AUVs. AUVs autonomously and actively move into the funnel-like docking station in this way. In this work, we address the recovery of AUVs by actively docking them with a funnel-like docking station carried by another slow-moving mother AUV.

The position sensing of docking stations is one of the most crucial modules for AUV recovery, no matter what forms of recovery are deployed. Three kinds of sensors are available for the position sensing of docking stations: (1) electromagnetic sensors, (2) acoustic sensors, and (3) optical sensors. The main concerns for AUV recovery are sensing range and resolution. Acoustic sensors have the longest sensing range, up to several kilometers, but the lowest resolution, with practically more than 1.5 m error [13]. Electromagnetic sensors have a better resolution than acoustic sensors, but much less range, about 30–50 m [17]. Optical sensors are superior in resolution, but inferior in propagation range. Their sensing range depends on water-body turbidity. It was verified in [18] that it is possible for optical sensors to guide AUV docking in the range of 10–15 m, even in turbid water. Landmarks are usually leveraged for optical-sensor-based visual navigation in recovering AUVs. They may either be passive or active landmarks. Passive landmarks are patterns generally drawn on boards. Active landmarks, such as light beacons, emit energy, having higher visibility compared to passive landmarks. LED beacons with 460-nm wavelength were used in our work due to their relatively good propagation in water. Positional errors of optical sensors, depending on the algorithms, could be less than 0.01 m [16,18]. Taking advantage of their positional accuracy, optical sensors are usually in charge of the final short-range stage precise positioning and combined with acoustic sensors [13,15,19], which are good in sensing range, but bad at resolution. Following this strategy, in this work, we also combined acoustic and optical sensors for AUV recovery. We focused on the development of visual-based AUV recovery (VBAR) algorithms using optical sensors in shallow water since many acoustic sensors for underwater positioning are now commercially available.

VBAR algorithms consist of a detection phase and pose-estimation phase. The detection phase localizes underwater landmarks in 2D images once landmarks appear in the view of the camera. Detection of landmarks in shallow water severely suffers from ambient light and nonuniform spreading [20]. Ambient light in open shallow water mainly comes from the Sun. The problem of ambient light in shallow water is more acute than in deep water. Previous methods for landmark detection in deep water are no longer effective in shallow water due to severe ambient light. Nonuniform landmark spreading is mainly caused by the camera’s view points. The luminance of landmarks close to the camera is stronger than those away from the camera. The developed methods so far have not dealt with the aforementioned problems particularly well. Some previous attempts took advantage of the luminance feature of landmarks for detection. An empirical fixed threshold was used for detection of landmarks by binarizing the underwater image in [1,21]. Binarizing underwater images with a fixed threshold is vulnerable to ambient light and nonuniform spreading. Underwater images were binarized with a fixed threshold within the region of interest (ROI), computed by a convolutional neural network in [16]. It overcomes ambient light to some extent. A histogram-based adaptive thresholding method was proposed for landmark detection in [20]. We show in Section 4 that this adaptive method is effective at landmark detection for short distances, but fails for remote distances. Remote detection of landmarks can provide AUVs with more time and space to adjust their pose for docking, which vastly increases docking success rate. AUVs cannot enormously adjust their pose within a short distance owing to their inherent mobility. We propose a Laplacian-of-Gaussian-based coarse-to-fine blockwise (LCB) method for the detection of underwater landmarks to overcome ambient light and nonuniform spreading.

The pose-estimation phase recovers the 3D relative position and orientation between landmarks and AUVs from detected landmarks on 2D images. Methods of pose estimation proposed for AUV recovery may be classified as based on points, lines, and curvature features. The pose was estimated by fitting lines and elliptical curves in [13,20], respectively. A points-based feature is the most commonly used due to its flexibility. A landmark is viewed as a feature point. A points-based feature was leveraged in [1,3,4,9,16,19,21] for pose estimation. Perspective-n-points (PnP) algorithms can accurately solve points-based pose estimation, but a drawback of PnP algorithms is that they fail in estimating poses in cases where not all predefined landmarks are detected, namely incomplete observations. However, an incomplete observation is highly possible in long-duration underwater tasks and may be caused by broken landmarks and biofouling coverage on landmarks in practice. In order to solve this problem, we propose a novel pose-estimation method for incomplete observations in AUV recovery. To the best of our knowledge, we are the first to solve the problem of pose estimation for incomplete observations in PnP-based VBAR.

Our contributions are four-fold:

- Detection of landmarks in shallow water suffers from ambient light and nonuniform spreading. In order to overcome them, we propose an LCB method for the detection of underwater landmarks.
- Incomplete observations of all predefined landmarks that may be caused by broken landmarks and biofouling coverage comprise a practical problem in pose estimation for recovering AUVs in long-duration missions. A novel pose-estimation method for incomplete observations in AUV recovery is proposed in this work. To the best of our knowledge, the proposed method is the first points-based method for pose estimation in VBAR algorithms.
- Field experiments were performed to evaluate the proposed LCB method and the pose-estimation method.
- We provide an underwater active landmark dataset that was collected in our field experiments. The 2D location of each landmark was manually labeled for each image. The dataset can help researchers develop and validate their underwater vision-based recovery or docking algorithms in the absence of related underwater infrastructures.

## 2. System Overview

**Figure 1:**
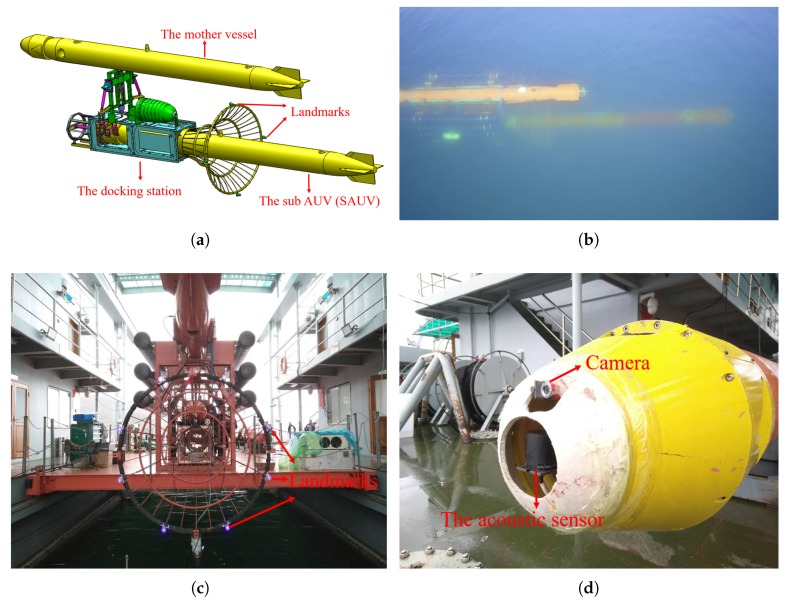
Autonomous underwater vehicle (AUV) recovery illustrated by (**a**) the simulation model and (**b**) field experiments, captured by an unmanned aerial vehicle. (**c**) Landmarks mounted on the docking-station rim. (**d**) Camera and acoustic sensor on the AUV head.

In this section, we introduce an overview of our recovery system. We illustrate the process of recovery in Figure 1a,b. The recovery system consisted of a mother vessel and a sub-AUV (SAUV) for recovery. The SAUV was attached to the docking station, physically and autonomously carried by the mother vessel, for recovery by employing underwater docking. The docking station was rigidly fixed to the underbelly of the mother vessel. The entrance of the docking station was funnel-like, 2 m in diameter, and equipped with several active landmarks mounted around its rim, as shown in Figure 1c. These landmarks were used for detecting and computing the position of the docking station, whose details are given in Section 3. Blue LEDs with a 460-nm wavelength were employed as our landmarks due to their good propagation in water. We utilized another AUV as the mother vessel in our system. The mother vessel could also be in other forms, such as unmanned surface vehicles (USVs).

The SAUV was a torpedo-shaped vehicle that was 384 mm in diameter and 5486 mm in length. It was equipped with a control computer, Doppler velocity log (DVL), inertial measurement unit (IMU), GPS, battery units, and motors. An acoustic sensor, *Evologics S2C R*, was used for rough long-range positioning, and our vision-based underwater-docking module for short-range precision guidance was installed in the SAUV, as shown in Figure 1d. A $NanoSeaCam^{®}$ monocular camera with a frame rate of 20 fps was rigidly mounted on the SAUV head for capturing underwater images. The captured images were fed into an embedded computer, Jetson TX2, for detecting the docking station and computing its pose (Section 3). Jetson TX2 is a power-efficient embedded computer with a 6-core CPU, 8 Gb RAM, and a 256-core GPU. The computed pose information was further transmitted to the SAUV controller for short-range precision navigation.

## 3. Underwater-Docking Recovery Algorithm

Our proposed underwater-docking algorithm for recovery consisted of two phases. The first phase was landmark detection, and the second was pose estimation. In the detection phase, the detector localizes 2D landmark coordinates on images once the landmarks appear in the field of view of the camera. The pose-estimation phase recovers the relative 3D pose, including position and orientation, between the docking station and SAUV. The estimated pose is then transmitted to the control computer of the SAUV for short-range precision navigation.

### 3.1. Landmark Detection

Landmarks in our task were LEDs actively emitting energy. The main problems in the detection of landmarks are nonuniform spreading and complex ambient light. In order to overcome these problems, we proposed an LCB method for landmark detection. In the coarse detection phase, we deployed a convolutional neural network, DoNN, proposed in [16], to detect the bounding box of the landmarks. Then, we proposed a Laplacian-of-Gaussian (LoG) filter that utilizes blob features to overcome ambient light. Blockwise segmentation on filtered images was also performed to deal with nonuniform spreading. We show that the proposed detector outperformed state-of-the-art methods in the remote detection of landmarks in Section 4.

**Coarse detection phase.** We leveraged a convolutional neural network, DoNN, proposed in [16], to detect the landmark bounding box. DoNN showed superior performance in robust bounding box detection of underwater landmarks in various underwater environments in [16]. Hence, DoNN was employed to detect the bounding box of all underwater landmarks, which is also known as coarse detection. We also validated the importance of coarse detection performed by DoNN by comparing the performance in Table 2. Rows 3 and 5 in the table show that the method leveraging coarse detection significantly outperformed the method that did not take advantage of coarse detection. Instead of first detecting the bounding box of all the landmarks, an alternative solution is to detect each individual landmark directly. However, the solution is not as stable and robust as detecting the pattern formed by all the landmarks. There are many noisy luminaries that are similar to landmarks in terms of their appearance in water. Noisy luminaries may come from other light sources from the external environment and mirror images of landmarks reflected from the water surface due to total internal reflection, as given in [1,16]. If detection is performed on individual landmarks, noisy luminaries become potential detection results. Instead, the pattern formed by all landmarks represent the concept of the docking station, which is more stable and robust for detection. For the above reasons, the bounding box of all landmarks was detected in our work.

For the sake of completeness, we briefly introduce DoNN. DoNN contains nine convolution layers and seven pooling layers. It maps captured underwater images to a vector v={x,y,w,h,s}, DoNN:(I)↦v. *I* denotes the captured underwater image. {x,y}, {w,h} represent the position and size of the predicted bounding box, respectively. *s* is the confidence score predicted by DoNN for the bounding box. DoNN first divides the input underwater image into G×G grids and then predicts multiple bounding boxes encoded by {x,y,w,h} along with the confidence score *s* for each grid. Finally, the bounding box with the highest confidence score *s* is selected as the final predicted bounding box. To summarize, the phase of coarse detection outputs the predicted confidence score and landmark bounding box.

**Figure 2:**
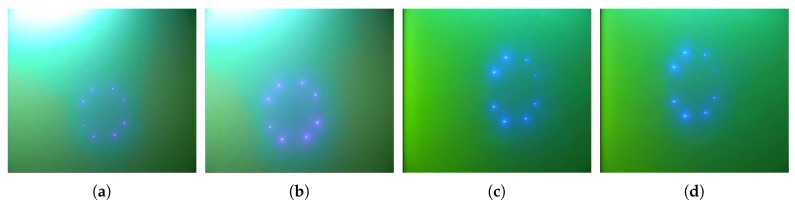
(**a**,**b**) Ambient light in AUV recovery in shallow water; (**c**,**d**) nonuniform spreading of landmarks in AUV recovery.

**LoG filter** After coarse detection by DoNN, the bounding box of landmarks is localized in the image. The following processes were performed to localize landmarks within the image patch given by the bounding box. LEDs actively emitting energy were designed as our landmarks due to their good propagation in water. Since such landmarks are quite bright, most previous works proposed to detect and extract landmarks by taking advantage of the luminance features of landmarks. Methods based on luminance features suffer from ambient light, and the problem becomes more acute in shallow water, as shown in Figure 2a,b. Besides luminance, landmarks have blob-like structures that were neglected by previous works. Blob features were employed to overcome the interference of strong ambient light in shallow water.

The LoG filter is a differential blob detector. It is defined as:

(1) $$LoG\left(x,y\right)≜\nabla^{2}G_{\sigma }\left(x,y\right)=\frac{\partial^{2}}{\partial x^{2}}G_{\sigma }\left(x,y\right)+\frac{\partial^{2}}{\partial y^{2}}G_{\sigma }\left(x,y\right)=\frac{x^{2}+y^{2}-2\sigma^{2}}{\pi \sigma^{4}}exp\left(-\frac{x^{2}+y^{2}}{2\sigma^{2}}\right),$$

where σ is a standard deviation of Gaussian filter $G_{\sigma }\left(x,y\right)$, $\nabla^{2}$ is the Laplacian operator, and (x,y) are image coordinates. Images filtered by LoG can be implemented by first convolving the image with a Gaussian filter $G_{\sigma }\left(x,y\right)$ and then applying the Laplacian operator $\nabla^{2}$ to the image filtered by $G_{\sigma }\left(x,y\right)$, since:

(2) $$f^{\prime }\left(x,y\right)=\nabla^{2}\left(G_{\sigma }\left(x,y\right)*f\left(x,y\right)\right)=\left(\nabla^{2}G_{\sigma }\left(x,y\right)\right)*f\left(x,y\right)=LoG\left(x,y\right)*f\left(x,y\right),$$

where f(x,y) is the pixel value of the image at (x,y) and $G_{\sigma }\left(x,y\right)$ is a Gaussian kernel defined as:

(3) $$G_{\sigma }\left(x,y\right)=\frac{1}{\sqrt{2\pi \sigma^{2}}}exp\left(-\frac{x^{2}+y^{2}}{2\sigma^{2}}\right).$$

The Laplacian operator is very sensitive to noise since it is a second derivative measurement on the image. To counter this case, a Gaussian filter was leveraged to smooth the image and attenuate high-frequency noise.

**Figure 3:**
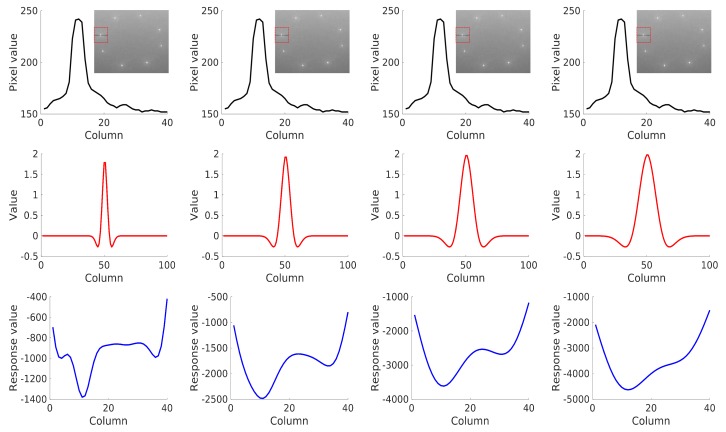
(First row) 1D pixel values along the black dotted line in the red bounding box. (Second row) Negatives of the Laplacian-of-Gaussian (LoG filters) with σ=3, σ=5, σ=7, and σ=9, from left to right. (Third row) Corresponding results obtained by convolving the original signal in the first row with the filter in the second row.

LoG filters obtain a strong response for dark blobs of σ extent on a light background or light blobs on a dark background when LoG filters convolve with images. The “3σ” rule indicates that 99% of the energy of a Gaussian is concentrated within three standard deviations of its mean. Hence, the standard deviation σ is usually computed as σ=(s−1)/3 to detect blobs of radius *s* [22]. Since the radius *s* of the blobs in our task is in the range of 10–25, the performance of our method with σ∈{3,5,7,9} was analyzed. Figure 3 shows the convolution of LoG filters (σ∈{3,5,7,9}) with landmarks in one dimension for ease of understanding. The response obtained its strongest value at the peak of the original signal. The response became smooth with the increment of σ.

**Blockwise segmentation** Nonuniform spreading is primarily due to the viewpoint of the camera, as illustrated in Figure 2c,d, posing great challenges to the extraction of landmarks from $f^{\prime }\left(x,y\right)$. Landmark images away from the camera are less bright than landmarks close to the camera, which results in a weak response in $f^{\prime }\left(x,y\right)$. To overcome this problem, we proposed to segment filtered images $f^{\prime }\left(x,y\right)$ in a blockwise manner. Filtered image $f^{\prime }\left(x,y\right)$ was first equally divided into four non-overlapping blocks so that the illumination of each block was approximately uniform. Then, we applied a global threshold method to each block for segmentation. Here, Otsu’s global segmentation method [23] was adopted for blockwise segmentation, which can work well since illumination is nearly uniform.

### 3.2. Pose Estimation

**Figure 4:**
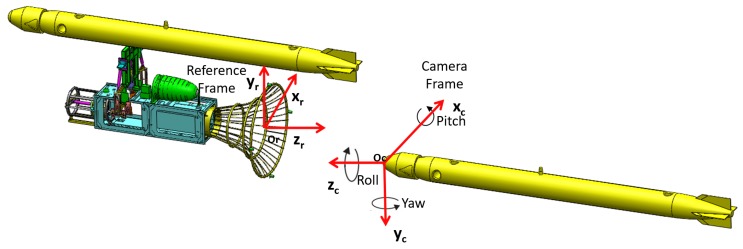
Coordinate frames in recovery.

The pose is defined as the relative 3D position, represented by X=(x,y,z), and orientation, represented by Euler angles (yaw, pitch, roll) between landmarks and the SAUV, as shown in Figure 4. Pose estimation is defined as the process of recovering the relative pose between landmarks and the SAUV from the 2D coordinates of landmarks localized by the LCB method. Landmarks in 2D underwater images were detected and localized in Section 3.1. In this section, we first introduce the method to recover the relative pose between landmarks and the SAUV in the case where all predefined landmarks are observed, namely complete observation. Then, our proposed method is provided for pose estimation in the case where not all predefined landmarks can be observed, namely incomplete observation. Pose estimation can be solved by the perspective-n-point (PnP) algorithm for the complete observation of landmarks. However, the PnP algorithm cannot work for the case of an incomplete observation. Incomplete observation of landmarks may be caused by broken landmarks and biofouling coverage on landmarks in practice. Pose estimation in the case of incomplete observations is imperative for the long-term use of unmanned systems. Previous works [1,16,21] that used the PnP algorithm required a complete observation of landmarks for pose estimation. The pose cannot be estimated in incomplete observations in these works. To our best knowledge, we are the first to solve the problem of pose estimation for the case of incomplete observations in points-based underwater recovery algorithms.

#### 3.2.1. Pose Estimation in the Case of Complete Observations

In order to estimate the pose between landmarks and the SAUV, three coordinate frames are first defined, as illustrated in Figure 4. The first coordinate frame is the camera coordinate frame, which is a 3D coordinate frame. Its origin is the optical center of the camera. Another coordinate frame is a reference frame attached to the docking station. Its origin resides in the geometric center of all landmarks. The third coordinate frame is the image coordinate frame, which is a 2D frame established on the image plane. The relative pose between landmarks and the SAUV is described by a transformation between the camera and reference coordinate frame. The relative pose is estimated by determining the transformation.

In the following, we detail the determination of the transformation between the camera and reference coordinate frame. Assume a pinhole camera is used; the transformation between the image and the camera coordinate frame can be expressed as a set of linear equations:

(4) $$\lambda \left[\begin{array}{c} u\\ v\\ 1 \end{array}\right]=\Lambda X_{c}=\left[\begin{array}{cccc} \phi_{x} & \gamma & \delta_{x} & 0\\ 0 & \phi_{y} & \delta_{y} & 0\\ 0 & 0 & 1 & 0 \end{array}\right]\left[\begin{array}{c} x_{c}\\ y_{c}\\ z_{c}\\ 1 \end{array}\right],$$

where λ is a scale factor, $\left(u,v\right)\in R^{3}$ is the coordinates in the image frame, $\left(x_{c},y_{c},z_{c}\right)\in R^{3}$ is the coordinates in the camera frame, and $\Lambda \in R^{3\times 4}$ is the intrinsic matrix of the inherent parameters of a camera, mapping 3D points in the camera frame to 2D points in the image. Λ contains five parameters. $\left(\delta_{x},\delta_{y}\right)\in R^{2}$ is the principal point, whose unit is pixels. $\phi_{x}\in R$ and $\phi_{y}\in R$ represent the scaling factor converting space metrics to pixel units. γ is the skew coefficient. The intrinsic matrix Λ of a camera can be calibrated before using the camera for pose estimation.

A matrix, called extrinsic matrix *E*, relates the camera frame and the reference frame. It is defined by:

(5) $$\left[\begin{array}{c} x_{c}\\ y_{c}\\ z_{c}\\ 1 \end{array}\right]=EX_{r}=\left[\begin{array}{cc} R & T\\ 0 & 1 \end{array}\right]\left[\begin{array}{c} x_{r}\\ y_{r}\\ z_{r}\\ 1 \end{array}\right],$$

where $E\in R^{4\times 4}$ is the extrinsic matrix, $\left(x_{r},y_{r},z_{r}\right)\in R^{3}$ are the coordinates of the reference frame, and *R* and *T* are the rotation and translation matrix, respectively. The relative 3D position and orientation of the relative pose can be obtained from *T* and *R*, respectively.

Combining Equations (4) and (5), the relationship between the image frame and the reference frame is:

(6) $$\lambda x=\lambda \left[\begin{array}{c} u\\ v\\ 1 \end{array}\right]=\Lambda E\left[\begin{array}{c} x_{r}\\ y_{r}\\ z_{r}\\ 1 \end{array}\right]=\Lambda Ew=\Omega w.$$

Given a known intrinsic matrix Λ, *n* 3D points $w=(x_{r},y_{r},z_{r})$ in the reference frame, and their corresponding projections x=(u,v) in the image, rotation matrix *R* and translation matrix *T* can be computed. This problem is known as the PnP problem. A unique solution can be obtained when n≥4. Given *n* point correspondences, Equation (6) can be solved using direct linear method “DLT” [24], which is a noniterative method. Noniterative methods are efficient, but less accurate and stable in the presence of noise. Iterative methods enhance solution accuracy by minimizing a nonlinear error function, such as image space error [25,26] and object space error [27]. However, the computational cost of the iterative methods is usually high. RPnP [28] is a noniterative method that is as accurate as iterative algorithms with much less computational time. Hence, RPnP was employed in our work for pose estimation. The main idea of RPnP consists of three steps.

**Figure 5:**
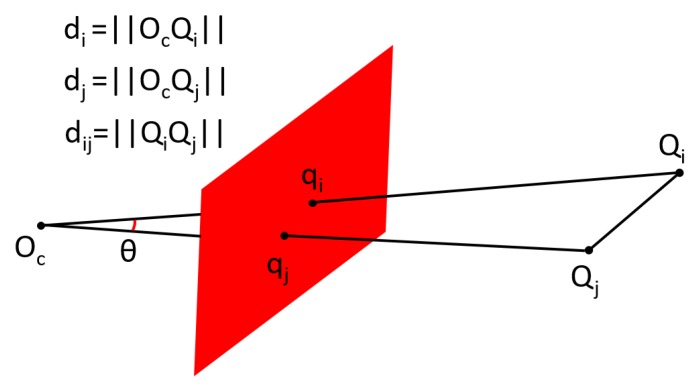
Constraint given by Equation (7).

Firstly, *n* reference points are divided into (n−2) three-point subsets. According to the law of cosines, each point pair $\left(Q_{i},Q_{j}\right)$ in the subset gives constraint:

(7) $$f_{ij}\left(d_{i},d_{j}\right)=d_{i}^{2}+d_{j}^{2}-2d_{i}d_{j}cos\theta_{ij}-d_{ij}^{2}=0,$$

where $d_{i}=\left|\right|O_{c}Q_{i}\left|\right|$, $d_{j}=\left|\right|O_{c}Q_{j}\left|\right|$, $d_{ij}=\left|\right|Q_{i}Q_{j}\left|\right|$ and $\theta_{ij}$ is the view angle, as illustrated in Figure 5. $Q_{i}$ and $Q_{j}$ are 3D reference points. $q_{i}$ and $q_{j}$ are the projection of $Q_{i}$ and $Q_{j}$ on the image plane, respectively. Based on Equation (7), we obtain the following polynomial system:

(8) $$\left\{\begin{array}{c} f_{12}\left(d_{1},d_{2}\right)=0,\\ f_{13}\left(d_{1},d_{3}\right)=0,\\ f_{23}\left(d_{2},d_{3}\right)=0, \end{array}\right.$$

for each subset. Equation (8) is then converted to a fourth-degree polynomial:

(9) $$g_{k}\left(x\right)=a_{1}x^{4}+a_{2}x^{3}+a_{3}x^{2}+a_{2}x+a_{1},\;\forall k=1,2,...,\left(n-2\right)$$

for the $k^{\mathrm{th}}$ subset.

Second, a cost function $G=\sum_{k=1}^{n-2}g_{k}^{2}\left(x\right)$ is defined as the square sum of the polynomials in Equation (9). It was demonstrated in [28] that cost function *G* has four minima at most.

Finally, the minima of *G* were explored by finding the roots of its first derivative $G^{\prime }=\sum_{k=1}^{n-2}g_{k}\left(x\right)g_{k}^{\prime }\left(x\right)=0$, which is a seven-degree polynomial. Rotation matrix *R* and translation matrix *T* were computed from each local minimum, and the result with the least reprojection residual was selected as the final solution.

Rotation matrix *R* was then further converted to Euler angles represented by (yaw, pitch, roll). Translation matrix *T* denotes the coordinates of point $O_{r}$ in the camera frame. The Euler angles and translation matrix *T* were passed to the SAUV controller for navigation.

#### 3.2.2. Pose Estimation in the Case of Incomplete Observations

Pose estimation can be solved by normal PnP algorithms using 2D-3D-point correspondences in the case of complete observations. However, PnP algorithms cannot give correct solutions if some landmarks are not observed without knowing which landmarks are missing. In this subsection, we propose a method to estimate pose in the case of incomplete observations by minimizing the reprojection error.

**Figure 6:**
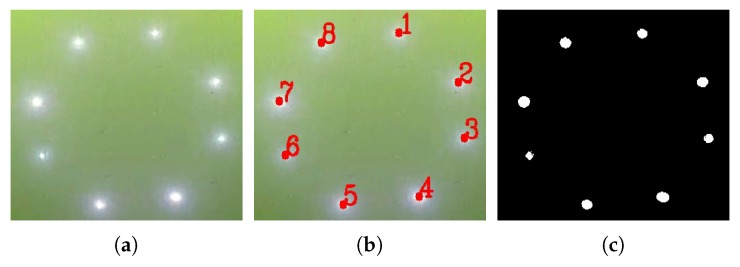
(**a**) Original image patch obtained by the coarse detection phase. (**b**) Definition of identification for (**a**). (**c**) Binary image of (**a**), computed by our proposed Laplacian-of-Gaussian-based coarse-to-fine blockwise (LCB) method, introduced in Section 3.1.

In order to estimate pose in the case of incomplete observations, the method first identifies which landmarks are missing. Then, the normal PnP algorithm can be used to compute the pose after identifying missing landmarks. Each landmark was numbered as its identification as shown in Figure 6a. The identification of all detected landmarks is denoted by a vector $i_{m}=\left\{1,2,3,\cdots ,m\right\}$, where *m* is the number of predefined landmarks. For example, vector $i_{8}=\left\{1,2,3,\cdots ,7\right\}$ means that there are eight landmarks in total mounted on the docking station, but the eighth landmark cannot be observed due to some reasons. Computing identification of landmarks $i_{m}$ is not hard in complete observations. Take the configuration of landmarks in Figure 6b as an example. The configuration consists of eight landmarks. The upper-right connected component in the binary images obtained by the detection phase corresponds to the first landmark. The upper-left connected component corresponds to the eighth landmark. The rest can be done in the same manner. The 2D-3D-point correspondences are gained in this way in the case of complete observations. However, this method does not work for the identification of landmarks in the case of incomplete observations. We propose to identify landmarks in the case of incomplete observations by finding an identification vector $i_{m}$ that minimizes the reprojection error, that is,

(10) $$argmin_{i_{m}}\sum_{j=1}^{k}{\left(\Omega_{i_{m}}w_{j}-x_{j}^{i_{m}}\right)}^{2},$$

where k(4≤k≤m) denotes the number of landmarks detected in the detection phase, $w_{j}$ are the 3D coordinates of the *j*th landmark defined in Equation (6), $x_{j}^{im}$ are the 2D coordinates of the *j*th landmarks determined by $i_{m}$, and $\Omega_{i_{m}}$ is the projection matrix in Equation (6), which is computed by using $w_{j}$ and $x_{j}^{i_{m}}$. To be specific, *k* is computed by analyzing connected components in the binary image obtained in the detection phase. Then, vector $i_{m}$ that minimizes the reprojection error is searched in the space of *k* landmarks. To search for the optimal $i_{m}$, $x_{j}^{i_{m}}$ was first computed for each $i_{m}$. Then, $\Omega_{i_{m}}$ was computed using the corresponding $x_{j}^{i_{m}}$ and $w_{j}$. Next, $w_{j}$ was projected to the image plane by $\Omega_{i_{m}}$, and the corresponding reprojection error was computed. Finally, the $i_{m}$ with the minimal reprojection error was the optimal $i_{m}$. After obtaining the optimal $i_{m}$, the relative pose can be computed using normal PnP algorithms.

## 4. Experiment Results

In this section, we give the experiment results of our proposed vision-based underwater recovery framework for AUVs. We first show that our proposed detection method outperformed the state-of-art method in landmark remote detection. Next, the LCB method is demonstrated on an underwater-docking dataset. Finally, we jointly validate our proposed method for detection and pose estimation in field experiments.

### 4.1. Experiment Detection Results

#### 4.1.1. Comparison of Detection: Algorithm Performance

**Figure 7:**
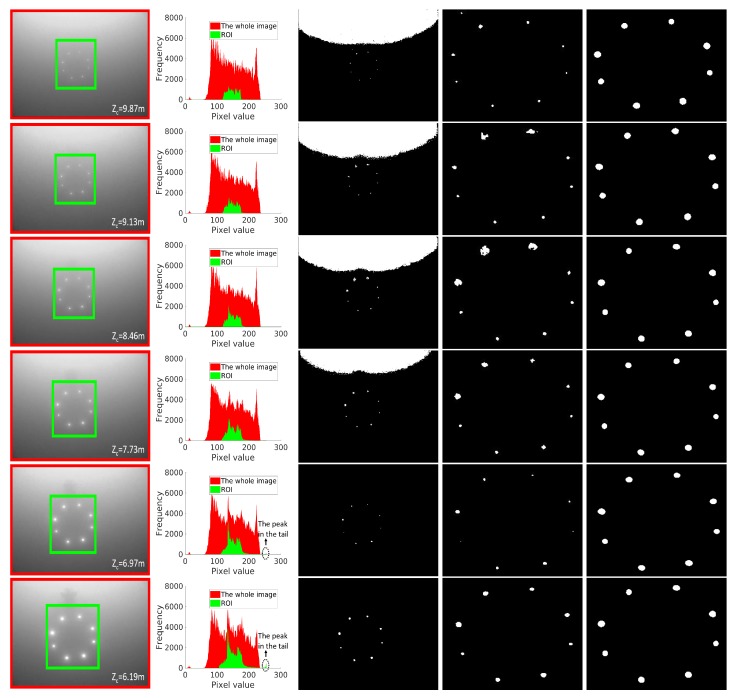
(First column) Distance-ordered landmark images captured in a run of underwater recycling. The distance between camera and docking station in the $z_{c}$ direction is shown in the lower right corner of each image. The green rectangle indicates the region of interest (ROI) predicted by the coarse detection phase. (Second column) Histograms of the whole image and ROI. The histogram in red corresponds to the whole image, indicated by the red rectangle. The histogram in green corresponds to the ROI, indicated by the green rectangle. (Third and fourth column) Detection results from applying HATS to the whole image (third column) and ROI (fourth column). HATS is only effective and used in the case where a peak exists in the tail (Rows 5 and 6). For cases in which a peak does not exist in the tail, a fixed threshold is used for segmentation. The fixed threshold was set to 180 in Rows 1–4. (Fifth column) Detection results of our proposed method (σ=5).

A histogram-based adaptive thresholding (HATS) method was proposed for landmark detection in [20]. We compare our proposed LCB method with HATS in this subsection. The comparison results are shown in Figure 7. Comparison images were consecutive frames taken in an AUV recovery task. HATS argues that landmark images contain a direct-path component and a glow component resulting from the scattering and absorption effects of the water body. The direct-path component carries much larger power than the glow component. Hence, the direct-path component corresponds to a peak in the tail of the histogram, as shown in Rows 5 and 6 of Figure 7. However, we found that HATS performed well in landmark detection at short distances, but failed at remote distances, because pixels of direct landmark components dominate the image at short distances, as shown in Rows 5 and 6 of Figure 7. The peak corresponding to the direct component was obvious in the tail of the histogram, but this was not the case at remote distances. The direct component of a landmark may only correspond to several pixels in the image at remote distances, resulting in a flat tail in the histogram, as illustrated in Rows 1–4 of Figure 7. In this case, landmarks cannot be detected using HATS, whereas remote detection is imperative for recovery since the remote detection of landmarks can provide the SAUV with more time and space to adjust its pose for docking, which vastly increases the docking success rate. Figure 7 shows that the LCB algorithm was effective for landmark detection at both short and remote distances, outperforming the state-of-the-art HATS method.

We further compared the LCB and HATS methods on a dataset, namely $D_{recovery}$ (Publicly available at https://drive.google.com/open?id=1m3OIQmtYUEwRmA-rN_Xg_eBYaUM1rarz), which contains 2306 images of active underwater landmarks. $D_{recovery}$ was collected in shallow-water field experiments with the landmarks and docking station deployed in [16]. Eight landmarks (m=8) were mounted on the docking station. The coordinates of each landmark in the 2D image were manually labeled as the ground truth in $D_{recovery}$. Detection was regarded as a failed detection if the number of detected landmarks was not equal to the number of predefined landmarks (k≠m). This can be regarded as an incomplete observation and solved by our proposed pose-estimation method if the number of detected landmarks is less than the number of predefined landmarks (4≤k<m). The Euclidean distance of each landmark between the location detected by the LCB algorithm and ground truth was computed for measuring location error if k=m. The location error was evaluated, as it significantly affected the accuracy of the pose-estimation algorithm given the known intrinsic matrix Λ and *n* 3D points $w=(x_{r},y_{r},z_{r})$ in the reference frame, as illustrated by (6). The comparison results are shown in Table 1. For the methods in Rows 1 and 2 in Table 1, the threshold was computed using HATS if a peak existed in the tail of the histogram. Otherwise, a fixed threshold was used. The first line shows results in which HATS was applied to the whole image. Both the successful detection and location errors were very poor. This was primarily due to the ambient light illustrated in the third column of Figure 7. HATS was applied to the ROI detected in the coarse detection phase in the second row of Table 1. Both failed detection and location error were improved thanks to coarse detection. Location error was 1.91, which is acceptable for successful detection, but the number of failed detections was very high. This was because HATS only works at short distances, as illustrated in the beginning of this subsection. The third row of Table 1 shows the results of our proposed LCB method. It performed much better than the two previous methods, in both the number of failed detection and location errors.

#### 4.1.2. Detection-Performance Analysis

**Figure 8:**
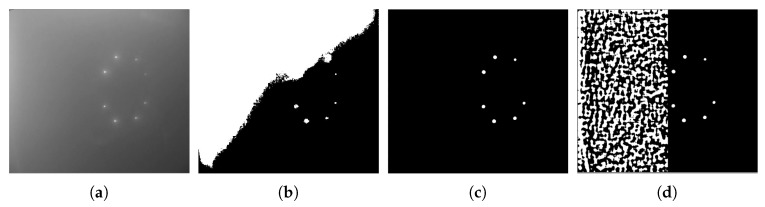
(**a**) Original image. (**b**–**d**) Results obtained by using the method in the first, second, and third row of Table 2, respectively.

The LCB method consists of three phases: (1) coarse detection, (2) LoG filtering, and (3) blockwise segmentation, as described in Section 3.1. In this section, we discuss the contribution of each phase to the detection performance of active underwater landmarks. Analysis was performed on $D_{recovery}$. Analysis results are shown in Table 2. The first row of Table 2 shows the detection performance by globally applying Otsu’s method to the image. Otsu’s method failed in the detection of all images in $D_{recovery}$ because it is severely affected by ambient light. Otsu’s method assumes that there are two classes of pixels that follow a bimodal histogram in images and finds a threshold to minimize the intraclass variance. Otsu’s method tends to segment the region close to the light source instead of individual landmarks, as shown in Figure 8b. The method globally applying LoG and then using Otsu’s method on the filtered image in the second row of Table 2 performed better than Otsu’s method in terms of resistance to ambient light, but poorly in nonuniform spreading, as seen in Figure 8c. Applying Otsu’s method to the image filtered by LoG block-wisely (Row 3 in Table 2) still separated the image in blocks that did not contain landmarks into two classes, introducing much blob noise, as shown in Figure 8d. Subsequently, we discuss the performance of methods with coarse detection (Rows 4–8). Comparing the method in the second and fourth rows, the number of failed detections was decreased by 75 images by coarse detection. Comparing the method in the fourth and fifth rows, the number of failed detections dramatically dropped, from 322 to 9 images, by adopting blockwise segmentation. We compare the performance of methods with various σ in Rows 5–8 of Table 2. Results showed that the method with σ=3 had the worst performance, and other methods (σ=5,7,9) performed almost the same. The poor performance of the method with σ=3 was primarily due to a small standard deviation in the LoG filter being sensitive to noise.

### 4.2. Pose-Estimation Algorithm Analysis

**Figure 9:**
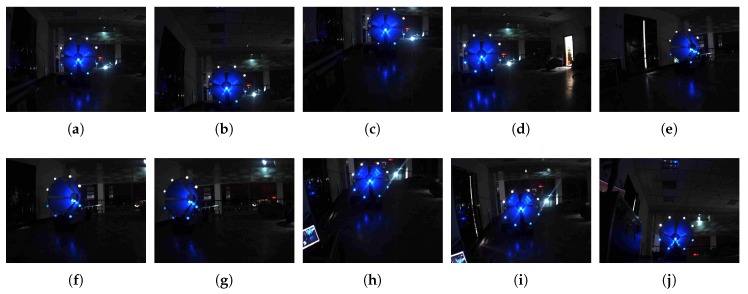
Landmarks for pose estimation in ground experiments.

In this subsection, we analyze the accuracy of the pose-estimation algorithm and its sensitivity to the location error introduced by the detection in 2D images. Since it is very hard to obtain the underwater ground truth of the relative pose between the SAUV and the docking station, accuracy was evaluated by ground experiments. In the ground experiments, a docking station of one meter in diameter was kept stationary on the ground, and a camera captured landmark images mounted on the docking station with various positions and orientations. To estimate the relative pose, the 2D location of each landmark in the 2D images was first annotated. Next, the relative pose using the pose-estimation algorithm was computed to assess accuracy in the absence of location error. Then, two levels of Gaussian noise, with standard deviations $\sigma^{\prime }=3$ and $\sigma^{\prime }=5$, were added to the annotated 2D location of each landmark to evaluate the accuracy of the pose-estimation algorithm in the presence of location errors in different degrees. Note that the intrinsic parameters of the camera used in the ground experiments were different from those used in water due to a different optical medium. Figure 9 and Table 3 (Part of the data in the table was published in our previous work [16].) show images for estimating the pose and corresponding results, respectively. Table 3 gives the mean estimation results averaged over 1000 trials for each degree of noise. It is shown in Table 3 that the mean orientation error was 1.823 degrees, and the mean position error was 6.306 mm in the absence of location error, that is $\sigma^{\prime }=0$. The mean orientation and position error increased to 2.168 degrees and 7.039 mm, respectively, as the noise level went to $\sigma^{\prime }=3$. With $\sigma^{\prime }=5$, the mean orientation and position error became 2.770 degrees and 9.818 mm, respectively. The aforementioned results validate the accuracy of the pose-estimation algorithm and its robustness to noise.

### 4.3. Field Experiments

**Figure 10:**
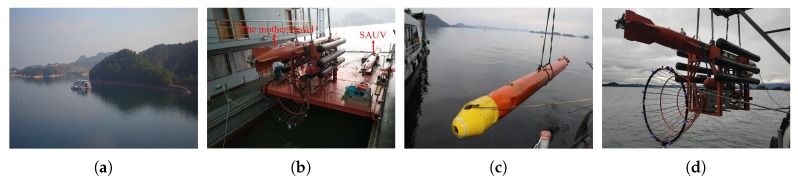
(**a**) Experiment ship in field experiments. (**b**–**d**) Mother vessel and sub-AUV (SAUV) in field experiments.

**Figure 11:**
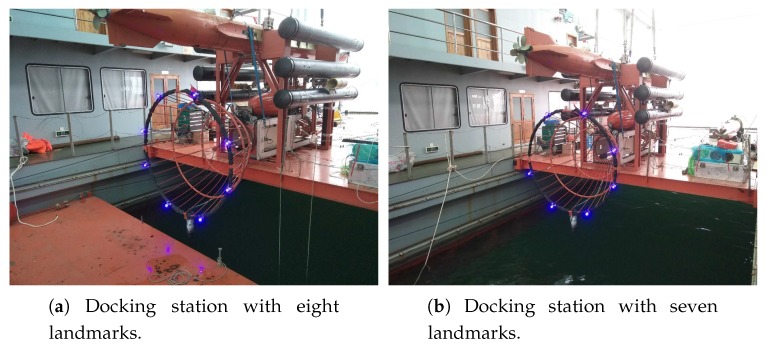
Docking stations with eight and seven landmarks.

We demonstrate our proposed LCB method and pose-estimation method by the automated recovery of AUVs in field experiments. Field experiments were conducted on an experimental ship, as shown in Figure 10a, anchored in Qiandao Lake, China, using systems given in Section 2. The method in the fifth row of Table 2 was employed for landmark detection. In order to validate our proposed pose-estimation method for both complete and incomplete observations, field experiments were performed with two configurations of landmarks. One was complete observations, that is all landmarks could be observed (k=m=8), as shown in Figure 11a. The other was to simulate incomplete observations (4≤k<m). To this end, a landmark was removed in advance, as shown in Figure 11b, but without letting the pose-estimation algorithm know this prior information.

**Figure 12:**
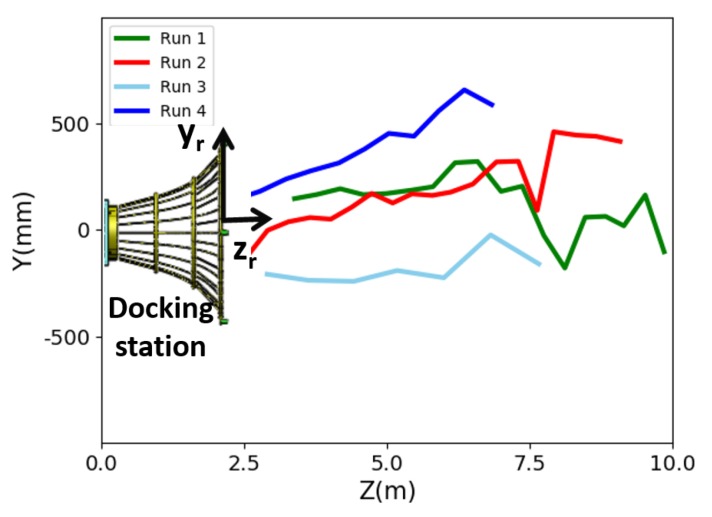
Trajectories of four recycling runs.

**Figure 13:**
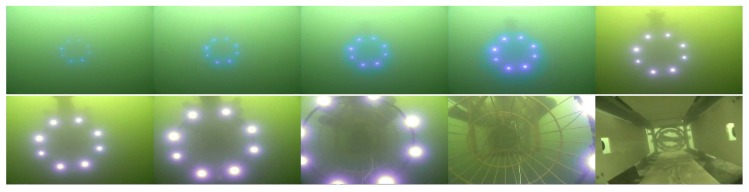
Images of the recycling process of Run 1.

**Figure 14:**
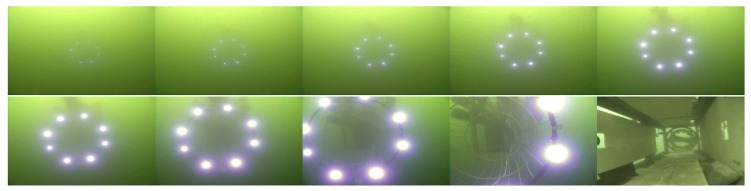
Images of the recycling process of Run 2.

**Figure 15:**
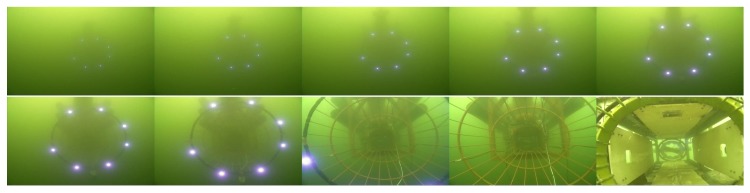
Images of the recycling process of Run 3.

**Figure 16:**
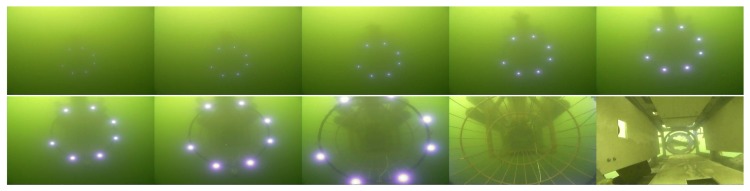
Images of the recycling process of Run 4.

Both the mother vessel (see Figure 10b,d) and the SAUV (see Figure 10b,c) started running at a random position with a random heading. The distance between them was about 500 m. The mother vessel ran near the water surface. Both acoustic and optical sensors were combined for recovery in the field experiments. The acoustic sensor was utilized for rough navigation at a long distance. Once the SAUV received the navigation information provided by our proposed vision-based underwater recycling algorithm, the SAUV switched to using it for navigation. Two runs were performed for each landmark configuration. The SAUV was recovered successfully by the mother vessel in all of these runs. Figure 12 shows the trajectories of all four of these runs. Figure 13, Figure 14, Figure 15 and Figure 16 give the images of the recycling process. Runs 1 and 2 demonstrate the LCB algorithm and pose-estimation method in complete observations. We validated the LCB algorithm and pose-estimation method for the incomplete observation in Runs 3 and 4. The average running time of the proposed framework was 0.17 s per frame.

## 5. Conclusions

A vision-based framework was proposed in this work for recovering an AUV with another AUV in shallow water. The framework consisted of a detection phase and a pose-estimation phase. In order to overcome challenges resulting from ambient light and nonuniform spreading in shallow water, an LCB algorithm was developed for the detection of underwater landmarks. Experiments showed that the LCB algorithm outperformed the state-of-the-art method in terms of location error. For the pose-estimation phase, a novel pose-estimation method was proposed for the incomplete observation of all underwater landmarks, which is practical, but has been neglected by most previous works. Pose estimation for incomplete observations enables the recovery of AUVs, even if some landmarks are broken or occluded by biofouling, which is very likely in long-duration missions. In ground experiments, we observed that the mean position and orientation error were 1.823 degrees and 6.306 mm, respectively, in the absence of noise, and 2.770 degrees and 9.818 mm, respectively, in the presence of strong noise. Field experiments were performed to recover a sub-AUV by a mother vessel in a lake using our proposed framework. The field experiments validated that our developed framework was effective for recovering AUVs in shallow water. 

## Figures and Tables

**Table 1:** Performance comparison of histogram-based adaptive thresholding (HATS) and LCB methods.

Method	Number of Failed Detections	Location Errors (Pixel per Landmark)
1. HATS	1907	18.13
2. HATS (ROI)	1370	1.91
3. LCB (σ=5)	**9**	**1.68**

**Table 2:** Detection performance of various methods.

Method	Number of Failed Detections	Location Errors (Pixel per Landmark)
1. Otsu	2306	N/A
2. LoG (σ=5) + Otsu	397	1.83
3. LoG (σ=5) + Block-wise + Otsu	1715	7.39
4. Coarse detection + LoG (σ=5) + Otsu	322	1.64
5. Coarse detection + LoG (σ=5) + Blockwise + Otsu	**9**	1.68
6. Coarse detection + LoG (σ=3) + Blockwise + Otsu	26	1.67
7. Coarse detection + LoG (σ=7) + Blockwise + Otsu	10	1.70
8. Coarse detection + LoG (σ=9) + Blockwise + Otsu	11	1.70

**Table 3:** Ground pose-estimation performance. Results in the table correspond to the figures given in Figure 9.

No.	Ground Truth		$\sigma^{\prime }=0$		$\sigma^{\prime }=3$		$\sigma^{\prime }=5$	
	Orien. (deg)	Pos. (mm)	Orien. (deg)	Pos. (mm)	Orien. (deg)	Pos. (mm)	Orien. (deg)	Pos. (mm)
(a)	Yaw: 0.0	*x*: 99.2	1.231	10.366	0.960	99.916	0.293	98.862
	Pitch: 4.5	*y*: −73.6	2.809	−71.517	1.932	−72.216	2.069	−71.717
	Roll: −0.8	*z*: 3686.5	−1.273	3687.955	−1.258	3679.886	−1.244	3666.954
(b)	Yaw: 6.8	*x*: 98.1	2.115	96.559	1.196	95.318	1.127	95.288
	Pitch: −9.2	*y*: 839.8	−11.302	837.126	−11.911	834.394	−12.314	832.257
	Roll: −1.3	*z*: 3598.1	−2.142	3590.579	−2.135	3598.303	−2.128	3572.824
(c)	Yaw: 2.7	*x*: 435.8	−3.714	433.207	−4.509	431.473	−4.443	430.364
	Pitch: 12.2	*y*: −1036.2	16.827	−1029.845	16.277	−1028.301	16.198	−1024.642
	Roll: −1.2	*z*: 3553.8	−1.915	3538.244	−1.841	3531.964	−1.898	3518.536
(d)	Yaw: 18.9	*x*: −1212.8	22.741	−1213.576	22.503	−1211.537	22.265	−1206.448
	Pitch: −0.4	*y*: −167.8	3.573	−168.660	3.067	−168.734	3.050	−167.966
	Roll: −2.3	*z*: 3453.7	−2.042	3460.211	−2.237	3453.575	−2.183	3438.054
(e)	Yaw: −37.0	*x*: 515.5	−35.471	513.549	−33.238	518.459	−29.508	522.176
	Pitch: 1.9	*y*: −422.3	2.576	−427.395	2.563	−430.459	3.374	−430.230
	Roll: −2.9	*z*: 4098.4	−2.571	4088.068	−2.624	4115.437	−2.817	4120.387
(f)	Yaw: −28.3	*x*: −393.6	−27.256	−394.989	−26.977	−395.335	−25.288	−396.326
	Pitch: 2.5	*y*: −374.2	3.573	−374.406	3.472	−375.174	3.615	−377.715
	Roll: −1.2	*z*: 3307.9	−1.363	3304.232	−1.328	3309.443	−1.402	3329.823
(g)	Yaw: −12.0	*x*: −1296.5	−11.813	−1284.283	−11.366	−1285.865	−10.421	−1290.430
	Pitch: 2.6	*y*: −327.8	3.991	−323.670	4.033	−324.082	4.006	−325.703
	Roll: −1.9	*z*: 3102.0	−2.317	3073.298	−2.326	3078.568	−2.331	3090.632
(h)	Yaw: 10.4	*x*: −59.8	4.375	−62.959	4.274	−63.164	3.078	−63.927
	Pitch: 22.8	*y*: −966.9	25.979	−972.440	23.945	−973.179	22.672	−971.425
	Roll: −3.0	*z*: 3728.8	−3.420	3745.472	−3.508	3741.764	−3.665	3730.413
(i)	Yaw: 3.2	*x*: 12.3	−2.501	3.982	−2.122	20.593	10.690	23.763
	Pitch: 10.1	*y*: −46.3	8.912	−38.898	13.662	−38.762	7.017	−57.425
	Roll: 5.4	*z*: 3889.0	4.965	3881.185	6.749	3898.219	2.879	3899.503
(j)	Yaw: −1.4	*x*: 252.5	−5.338	259.961	−4.975	245.176	7.377	239.694
	Pitch: −17.6	*y*: 823.0	−16.601	831.225	−16.833	814.238	−19.823	810.849
	Roll: 1.8	*z*: 3954.4	1.733	3962.113	0.919	3945.519	4.324	3964.525
Mean Error	N/A	N/A	1.823	6.306	2.168	7.039	2.770	9.818
